# Influence of Fear of Pain and Coping Strategies on Health-Related Quality of Life and Patient-Anticipated Outcomes in Patients With Chronic Pain: Cross-Sectional Study Protocol

**DOI:** 10.2196/resprot.8205

**Published:** 2017-09-08

**Authors:** Manasi Murthy Mittinty, David S Brennan, Cameron L Randall, Daniel W McNeil, Murthy N Mittinty, Lisa Jamieson

**Affiliations:** ^1^ Australian Research Centre for Population Oral Health Adelaide Dental School The University of Adelaide Adelaide Australia; ^2^ Department of Psychology Eberly College of Arts and Sciences West Virginia University West Virginia United States; ^3^ Department of Dental Practice and Rural Health School of Dentistry West Virginia University West Virginia United States; ^4^ School of Public Health The University of Adelaide Adelaide Australia

**Keywords:** fear of pain, coping strategies, health-related quality of life, patient-anticipated outcomes, chronic pain

## Abstract

**Background:**

Fear of pain and coping strategies are emotional-behavioral responses to pain and are known to play an important role in the development and maintenance of pain. It is highly likely that fear of pain and coping strategies influence each other, potentially affecting the course of chronic pain. To our knowledge, the relationship between pain, fear of pain and coping strategies, and how they influence patient-anticipated outcomes and health-related quality of life, have not been investigated.

**Objective:**

The aims of this study are to test (1) if both fear of pain and/or coping strategies are sufficient causes for maintaining pain; and (2) whether fear of pain influences coping strategies and pain intensity. The study will also examine the impact of fear of pain and coping strategies on health-related quality of life and patient-anticipated outcomes.

**Methods:**

The cross-sectional study will be conducted using an online survey. The Fear of Pain Questionnaire-III (FPQ-III), the Brief Coping Inventory (COPE), and EuroQoL-5d (EQ-5D) validated questionnaires will be used to collect data. Information pertaining to demographic factors, pain-related factors, and patient-anticipated outcomes will also be collected. The study has ethics approval from the Human Research Ethics Committee of the University of Adelaide. Study participants will be individuals aged 18 years and above who are experiencing chronic pain (ie, pain lasting more than 6 months). Effect measure modification technique (EMMM) will be used to examine if fear of pain acts as a moderator or mediator between coping strategies and pain. Simple and multinomial logistic regression analysis will be used to examine the effect of fear of pain and coping strategies on health-related quality of life and patient-anticipated outcomes.

**Results:**

Recruitment began July 2017 and it is anticipated that data collection will be completed by October 2017. Findings from this study will help to extend our understanding of fear of pain and coping strategies, their interaction, and their impact on health-related quality of life and patient-anticipated outcomes.

**Conclusions:**

Fear of pain and coping strategies have significant influence on the experience of chronic pain and its course. This study will help enhance our understanding of the relationship between fear of pain and coping strategies, which may help in developing patient-centered care practices.

## Introduction

### Background

Chronic pain is a multifaceted, global health problem affecting nearly 1 in 5 individuals worldwide [1]. Its continuous presence has devastating effects on an individual’s personal, social, and work life [2]. Chronic pain invades everyday functioning, communications, and interactions. Its complex presentations and etiology still perplex healthcare professionals and scientists. To improve our understanding of the variations in presentation of chronic pain, it is essential to understand the close interactions between various relevant psychosocial and physiological processes [3].

As proposed by the Gate Control Theory [3], sensory information from afferent neurons to transmission cells in the spinal cord is moderated by the susbatantia gelationosa in the dorsal horn, which functions as a “pain gate.” This gate can affect the transmission of the nociceptive signal by opening or closing. It is postulated that this gate is modulated not only by input from supraspinal centers but also by the thoughts, feelings, and behaviors of the patient. In other words, negative view (eg, focusing on pain and non-constructive thoughts), feelings (eg, sadness, helplessness, anger, hopelessness), and behaviors (eg, inactivity, smoking, sleep deprivation) can potentially “open” the gate, while positive thoughts, feelings, and behaviors can prevent the gate from “opening” [3].

Fear is defined as an emotional and behavioral reaction [4] to immediate threat or a past distressing event, and fear of pain is explained as an emotional and behavioral response to stimuli [5] that are or are perceived to be painful. Fear of pain may cause aversion to movement or activity, or escape in response to noxious stimuli [5]. These responses are believed to evolve from catastrophic thoughts and beliefs and negative interpretations of the potentially painful stimulus [6]. The manner in which an individual responds or reacts to pain is often influenced by their fear of pain and not by the pain itself [7,8]. This maladaptive response is believed to precede persistent pain and disability through a fear-avoidance cycle [6] and plays a vital role in initiating, developing, and sustaining pain [9]. Similarly, coping is also described as a psychological response exhibited by individuals when managing stressful events such as chronic pain [10]. The coping strategies employed in response to a stressor significantly determines psychological adjustment and well-being of the individual [11]. Several distinct coping styles have been recognized, such as approach and avoidance coping [12], problem-focused and emotion-focused coping [13], situational and dispositional coping [14], and active and passive coping [15]. Although these styles are independent, they show influence on mood, anxiety, depression, behavior, and clinical outcomes [15,16]. The psychological shift observed in chronic pain patients can be guided by these coping strategies [17].

It is plausible that distinct emotional-behavioural responses can not only keep the pain gate “open” or “closed”, but can also likely influence and interact with each other affecting the course of chronic pain. The level of fear of pain could influence the choice of coping strategies applied by patients with chronic pain and conversely. It is also likely that coping strategies could act as mediator of the effect of fear of pain. To our knowledge, the relationship between pain, fear of pain and coping strategies has not previously been investigated. How these emotional-behavioral responses affect health-related quality of life and outcomes in patients with chronic pain also remains unclear. A better understanding of how these factors influence and interact with each other will help build knowledge that will ultimately facilitate improved care for chronic pain sufferers.

### Study Aim and Objectives

The aim of this study is to examine if there is an interaction between fear of pain and coping strategies and how this interaction influences patient-anticipated outcomes and health-related quality of life. We will also test the following hypotheses: (1) both fear of pain and coping strategies are sufficient causes for maintaining pain; and (2) fear of pain influences coping strategies and pain.

The specific objectives of this study are (1) to examine if fear of pain acts as a mediator or moderator between coping strategies and pain severity; (2) determine the common predictors of high fear of pain levels and passive coping strategies; (3) examine if fear of pain affects health-related quality of life and patient-anticipated outcomes reported by individuals experiencing chronic pain; and (4) examine if coping strategies affect health-related quality of life and patient-anticipated outcomes reported by individuals experiencing chronic pain.

## Methods

### Study Design

A cross-sectional design will be used to conduct this study. An online survey will be conducted as it is cost-effective and can reach a diverse group of populations regardless of geographical location. With an online survey, identical questions can be posed to all participants, which allows researchers to draw generalizable results tailored to patients with chronic pain.

Validated questionnaires will be used for this study, with the patient-anticipated outcomes assessment specifically designed after reviewing the literature and conducting discussions with researchers. The survey comprises the Fear of Pain Questionnaire-III (FPQ-III) [5], the Brief Coping Inventory (COPE) questionnaire [18], and EuroQol-5d (EQ-5D) [19]. The study will be administered via Survey Monkey. Ethical approval for this study was granted by the Human Research Ethics Committee of the University of Adelaide.

### Selection Criteria

Participants aged 18 years and above who are experiencing pain for more than 6 months and have good comprehension of the English language will be invited to participate in this study. Individuals younger than 18 years of age and experiencing chronic pain for less than 6 months will be ineligible.

### Recruitment

The study will be advertised on the University of Adelaide website. Health-related organizations and patient forums popularly accessed by patients with chronic pain will be approached for advertising the survey—both within Australia and internationally—using the networks of the study authors. Invitation for the study will inform the interested participants of our inclusion and exclusion criteria and assure potential participants of anonymity (no personal information will be collected during any stage of the survey) and voluntary participation. No direct contact will be established with potential participants.

### Consent

The invitation to take part in the study will include a Web link, which will take the participant to the information page of the survey. The information sheet will provide a description of the research team, purpose of the study, the type of questions asked in the survey, expected completion time, and contacts for grievances or feedback. At the bottom of the information sheet, the participant will be asked to “click” a box as their consent to participate in the study. Only participants providing their consent will be directed to the survey page.

### Data Storage and Handling

The data will be stored on a password-protected desktop computer located at the Australian Research Centre for Population Oral Health (ARCPOH). Because no information that could potentially identify the participant will be collected, the data will be completely anonymous. Any written information from the study will be stored on a password-controlled University of Adelaide computer, which itself is not accessible to the public. Only the listed authors will have access to the data collected.

### Sample Size

**Figure 1:**
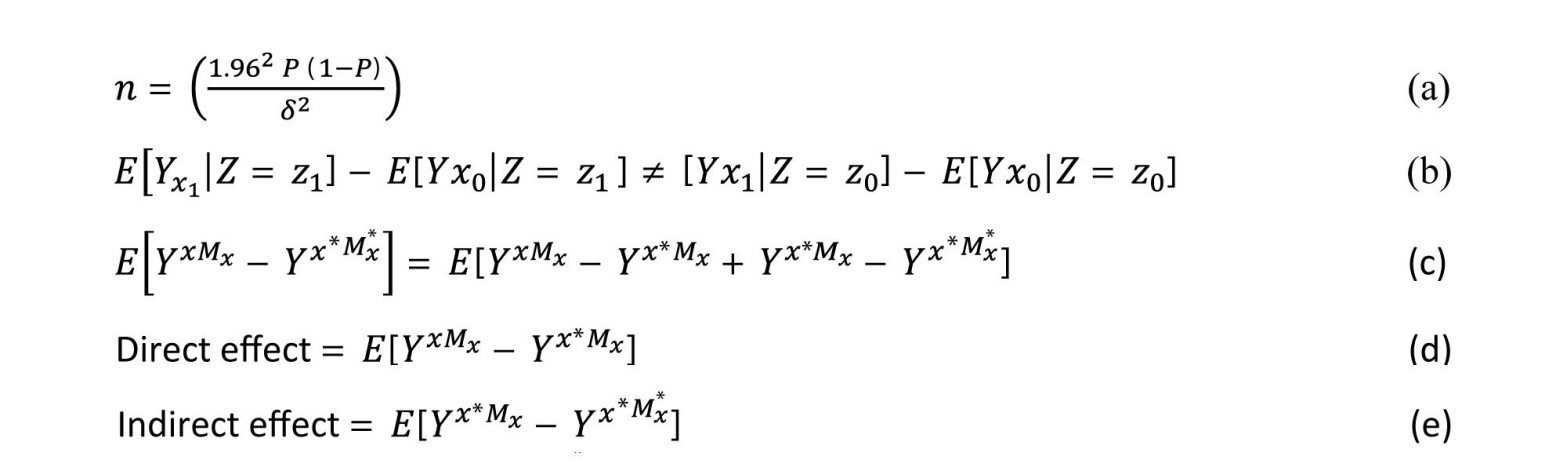
Study equations.

Following the simple random sampling approach, a sample size of 480 is estimated for 95% confidence interval, with *P* equal to .5 and delta of .05 (Figure 1, equation a), where n is sample size, P is the is the estimated population proportion, and 𝛿 is the precision of the estimate.

### Dependent Variables

#### Fear of Pain

The FPQ-III is a self-reporting scale that measures fear of severe pain, minor pain, and medical pain. The FPQ-III is a self-rating scale that measures fear of severe pain, minor pain, and medical pain. It uses a 5-point rating scale that measures fear across a range of situations which can trigger pain. The overall score (range 30 to 150) and subscale scores (range 10 to 50) will be calculated for every participant. The validity and reliability of FPQ-III has been confirmed through various studies [5,20-22].

#### Brief Coping Inventory Questionnaire

To examine the ways in which participants cope with everyday pain, the original version of the Brief COPE will be used [18]. The Brief COPE is based on the COPE inventory developed by Carver and colleagues [12]. It is a self-report scale measuring use of problem- and emotion-focused coping strategies across 14 different approaches (grouped into 14 different scales). Each item is scored using a 4-point Likert scale. As recommended by Carver et al [12], each scale will be recorded separately to determine its relationship with other variables. The validity and reliability of the Brief COPE questionnaire has been studied and confirmed by multiple studies performed in different countries and samples [23-26]. The Brief COPE was preferred over the original COPE questionnaire due to its concise format, which requires less time for completion, thus preventing participant fatigue.

### Independent Variables

#### Demographic Information

The survey will collect the following demographic information: age, sex, country of residence, metropolitan or non-metropolitan location, marital status, level of education, type and status of employment, and rating of family income in past 12 months (low, middle, and high income).

#### Information on Current Pain Problem

Information regarding most painful body part(s), diagnosis provided, and if the pain is related to an injury or accident with legal proceedings in process will also be collected.

#### European Quality of Life Questionnaire

The 5-level EQ-5D (EQ-5D-5L) [19] will be used to assess participants’ subjective assessment of their physical, mental, and social well-being. EQ-5D is a short report of health-related quality of life. It appraises movement, self-care, daily activities, pain/discomfort, anxiety/depression, and self-rated health state using a visual analogue scale (VAS). The questionnaire will be scored as recommended by the EQ-5D-5L committee. The reliability and validity of EQ-5D in various diseases has been shown by studies performed in different countries and populations [27-29].

#### Patient-Anticipated Outcomes

A detailed literature search was performed to understand the outcomes anticipated by individuals experiencing chronic pain. A total of 15 items were documented which were then grouped into the following domains: (1) pain-specific, (2) physiological, (3) social, (4) psychological, and (5) economical. Participants will be asked to select the 5 most expected outcomes from a list of outcomes (Textbox 1).

**Domains of clinical outcomes.**

Domain

- Pain-specific
  - Reduce pain
  - Reduce amount of daily medications
  - Self-management of pain or discomfort
- Physiological
  - Improve sleep and concentration
  - Improve physical functioning- more walking, exercise, movement and strength
  - Less tiredness and fatigue
- Social
  - Plan a holiday/travel
  - Socialize more with family and friends
  - Purse a hobby—cooking, gardening
- Psychological
  - Improve mood, less stress, anxiety and worry
  - Improve self-worth
  - Be optimistic about the future
- Economical
  - Go back to work/increase working hours and improve performance
  - Go back to studying
  - Improve financial earning

### Statistical Analysis

STATA statistical software [30] will be used for all statistical analyses.

#### Objective 1:

**Figure 2:**
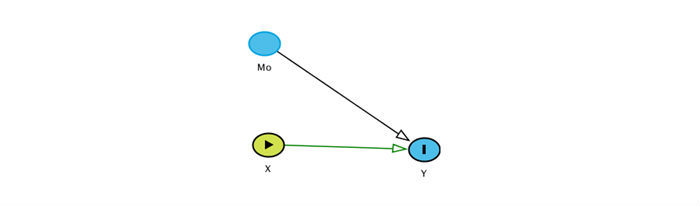
Hypothesis 1: fear of pain acts as a moderator between coping strategies and pain. X (exposure) is coping strategies; Y (outcome) is pain; Mo (moderator) is fear of pain.

**Figure 3:**
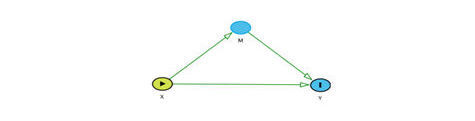
Hypothesis 2: fear of pain acts as a mediator between coping strategies and pain. x is exposure; y is outcome; M is mediator.

To examine if fear of pain acts as a mediator or moderator between coping strategies and pain severity, we will test 2 hypotheses in this objective. The first hypothesis is depicted in Figure 2 and the second hypothesis is depicted in Figure 3. In Figure 2, we hypothesize that both fear of pain and coping strategies are sufficient causes for maintaining pain. In other words, we hypothesize that fear of pain acts as a moderator between coping strategies and pain, where moderator is defined as the variable that has the potential to alter the strength of the causal relationship between exposure and outcome [31].

To examine if fear of pain acts as a moderator between coping strategies and pain we will use the effect measure modification technique (EMMM) as detailed by VandeerWeele [32] (Figure 1, equation b) where X is exposure, Y is outcome, and Z is another exposure which is not an effect of X. Thereby, the effect of X (exposure) on Y (outcome) may vary across the subpopulations of Z (second exposure (ie, for some levels of the exposure x_0_ and x_1_). Simply, the expected average change of the first exposure on the outcome is not constant within the realized values of the second exposure Z. Since it is sensitive to scale (eg, log [multiplicative] or linear additive), we will utilize the EMMM using both additive and multiplicative scales.

Alternatively, we also hypothesize that fear of pain influences coping strategies and pain (Figure 3). To test this second hypothesis we will conduct mediation analysis. From mediation analysis, we intend estimating the direct and indirect effects of fear of pain on pain. Direct and indirect effect effects will be estimated using the counterfactual theory. In counterfactual theory, we create the counter-to-the-fact scenarios and estimate the change in the outcome. Counterfactual theory is used as it allows us to estimate the marginal compared to the simple regressions, which only allows us to compute the conditional estimates [33]. In counterfactual theory, the direct estimate is the natural direct effect, and the indirect effect is the total indirect effect. The natural direct effect is the change in the potential outcome when the individual receives a treatment and the same individual does not receive treatment when the mediator is set to a counterfactual level. The total indirect effect is defined as the change in the potential outcome when treatment is set to the counterfactual level and the mediator is set to the observed and the counterfactual levels (Figure 1, equations c-e).

#### Objective 2

To determine the common predictors of high fear of pain and maladaptive coping strategies, we will conduct a simple logistic regression analysis because our outcome is measured as a binary variable.

#### Objective 3

Multinomial logistic regression modeling will be used to determine the effect of fear of pain on health-related quality of life and patient-anticipated outcomes Multinomial logistic regression modeling will allow us to predict the probability of categorical membership on the dependent variable based on multiple independent variables [27].

#### Objective 4

Univariate logistic regression modeling will be used to determine the effect of coping strategies on health-related quality of life and patient-anticipated outcomes. All analyses will be adjusted for confounders such as age, sex, residential location (ie, metro, non-metro), marital status, employment status, and education. The entire sample will be described using simple descriptive statistics such as means, proportions and variances.

### Dissemination

Findings from this study will be presented at conferences and public forums. The results will be published in peer-reviewed journals.

## Results

Participant recruitment and data collection began July 2017 and it is anticipated that all data will be collected by October 2017. The findings from this study will help to extend our understanding of fear of pain and coping strategies, their interaction, and their impact on health-related quality of life and patient-anticipated outcomes.

## Discussion

### Principal Findings

This study aims to extend our understanding of key emotional behavioral responses observed in chronic pain patients: fear of pain and coping strategies. While previous studies have assessed their effect on treatment outcomes, their impact on and interaction with each other has not previously been examined. Considering that both fear of pain and adaptive or maladaptive coping could co-exist in chronic pain patients, their association is predictable. However, we hypothesize that fear of pain, which can exist with or without a pain-causing event, can act as a mediator between the coping strategies and pain. It is anticipated that the study findings will help detect common predictors of fear of pain and adaptive versus maladaptive coping strategies, allowing healthcare professionals and researchers to build a management plan tailored to individual patient needs.

### Limitations

Causal inferences will not be able to be drawn from our findings due to the cross-sectional study design. As the study may potentially have participants from different countries, it may lack specificity.

### Conclusion

Enhancing our understanding of the interplay between fear of pain and coping strategies, and its effect on health-related quality of life and patient-anticipated outcomes, may increase understanding of how different psychosocial factors modify the course of chronic pain. It is also anticipated that the findings from this study may be helpful in developing patient-centered care strategies for chronic pain sufferers.

## Abbreviations

COPE: Coping Inventory
EMMM: effect measure modification technique
EQ-5D: EuroQol-5d
EQ-5D-5L: 5-level EQ-5D
FPQ-III: Fear of Pain Questionnaire-III
